# Macimorelin (AEZS-130)-Stimulated Growth Hormone (GH) Test: Validation of a Novel Oral Stimulation Test for the Diagnosis of Adult GH Deficiency

**DOI:** 10.1210/jc.2013-1157

**Published:** 2013-04-04

**Authors:** J. M. Garcia, R. Swerdloff, C. Wang, M. Kyle, M. Kipnes, B. M. K. Biller, D. Cook, K. C. J. Yuen, V. Bonert, A. Dobs, M. E. Molitch, G. R. Merriam

**Affiliations:** Michael E. DeBakey Veterans Affairs Medical Center/Baylor College of Medicine (J.M.G.), Houston, Texas 77030; Harbor-UCLA Medical Center and Los Angeles Biomedical Research Institute (R.S., C.W.), Torrance, California 90502; Radiant Research, Inc (M.Ky.), Chicago, Illinois 60654; DGD Clinic (M.Ki.), San Antonio, Texas 78229; Massachusetts General Hospital/Harvard Medical School (B.M.K.B.), Boston, Massachusetts 02215; Oregon Health and Science University (D.C., K.C.J.Y.), Portland, Oregon 97239; Cedars-Sinai Medical Center (V.B.), Los Angeles, California 90048; Johns Hopkins Medical Institutions (A.D.), Baltimore, Maryland 21205; Northwestern University Feinberg School of Medicine (M.E.M.), Chicago, Illinois 60611; and Veterans Affairs Puget Sound HCS/University of Washington (G.R.M.), Seattle and Tacoma, Washington 98108

## Abstract

**Context:**

In the absence of panhypopituitarism and low serum IGF-I levels, the diagnosis of adult GH deficiency (AGHD) requires confirmation with a GH stimulation test. Macimorelin is a novel, orally active ghrelin mimetic that stimulates GH secretion.

**Objective:**

The objective of the study was to determine the diagnostic efficacy and safety of macimorelin in AGHD.

**Design:**

This was a multicenter open-label study comparing the diagnostic accuracy of oral macimorelin with that of arginine+GHRH in AGHD patients and healthy, matched controls. After 43 AGHD patients and 10 controls were tested, the GHRH analog Geref Diagnostic [GHRH(1–29)NH_2_] became unavailable in the United States. The study was completed by testing 10 additional AGHD patients and 38 controls with macimorelin alone.

**Main Outcome Measure:**

Peak GH area under the receiver operating characteristic curve after macimorelin was measured.

**Results:**

Fifty AGHD subjects and 48 controls were evaluated. Peak GH levels in AGHD patients and controls after macimorelin were 2.36 ± 5.69 and 17.71 ± 19.11 ng/mL, respectively (*P* < .0001). With macimorelin, the receiver operating characteristic analysis yielded an optimal GH cut point of 2.7 ng/mL, with 82% sensitivity, 92% specificity, and 13% misclassification rate. For subjects receiving both tests, macimorelin showed discrimination comparable with arginine+GHRH (area under the receiver operating characteristic curve 0.99 vs 0.94, respectively, *P* = .29). Obesity (body mass index > 30 kg/m^2^) was present in 58% of subjects, and peak GH levels were inversely associated with body mass index in controls (r = −0.37, *P* = .01). Using the separate cut points of 6.8 ng/mL for nonobese and 2.7 for obese subjects reduced the misclassification rate to 11%. Only 1 drug-related serious adverse event, an asymptomatic QT interval prolongation on the electrocardiogram, was reported.

**Conclusion:**

Oral macimorelin is safe, convenient, and effective in diagnosing AGHD with accuracy comparable with the arginine+GHRH test.

Adults with a history of childhood-onset GH deficiency or with hypothalamic/pituitary disease, surgery, or irradiation to these areas, head trauma, or evidence of other pituitary hormone deficiencies are at risk for adult GH deficiency (AGHD). Because symptoms are usually nonspecific, in the absence of panhypopituitarism and low serum IGF-I levels, the diagnosis of AGHD requires biochemical confirmation with at least 1 GH stimulation test (1). The insulin tolerance test (ITT) is considered the gold standard test for AGHD, having a sensitivity of 96% and a specificity of 92% (1). However, because it induces hypoglycemia, the test is contraindicated in patients with coronary artery disease, seizures, and in the elderly (1). GHRH combined with arginine (arginine+GHRH) has been endorsed by several consensus guidelines (2–4) as the main alternative when the ITT is contraindicated, having a sensitivity of 95% and a specificity of 91% (1); but when GHRH analog (Geref Diagnostic; Serono Laboratories, Rockland, Massachusetts) was withdrawn in the United States in 2008, the need for an alternative to the ITT increased (2). The diagnosis of AGHD is important, given that the treatment of this condition, although expensive, has consistently shown improvements in body composition, exercise capacity, endothelial function, inflammatory biomarkers, bone mineral density, lipoprotein metabolism, and self-reported quality of life measures (5–10).

Ghrelin is the endogenous ligand for the GH secretagogue receptor [also called the ghrelin receptor (GHS-R1a)] (11, 12). Pharmacological treatment of rats and mice with ghrelin increases GH secretion, and *Ghsr*^−/−^ mice are refractory to the stimulatory effects of ghrelin on GH release, confirming that the GHS-R1a is the physiologically relevant ghrelin receptor mediating GH secretion (13).

Synthetic agonists of this receptor, known as ghrelin mimetics or GH secretagogues, are molecules that evoke dose-dependent increases in GH levels (14, 15). Macimorelin (formerly known as AEZS-130, ARD-07, and prior to that, EP-01572) is a novel GH secretagogue with good stability and oral bioavailability, which binds the GHS-R1a receptor and to pituitary and hypothalamic extracts with an affinity similar to ghrelin (16). In phase I clinical studies in healthy volunteers, macimorelin stimulated GH release in a dose-dependent manner, achieving maximum blood levels within 1 hour with good tolerability (17). The objective of this clinical trial was to determine the diagnostic efficacy and safety of macimorelin in the diagnosis of AGHD.

## Materials and Methods

### Study design

This study was conducted at 11 centers across the United States. The protocol was approved by the institutional review board at each institution. Patients were recruited between July 2007 and July 2011. The study was conducted in compliance with ethical principles that have their origin in the Declarations of Helsinki and its amendments and the International Conference on Harmonization Guideline for Good Clinical Practices. This multicenter, open-label study was originally designed as a crossover trial of oral macimorelin (0.5 mg/kg) vs GHRH (Geref Diagnostic; Serono) iv bolus of 1 μg/kg + arginine (Ar-Gine; Pfizer, New York, New York) iv infusion of 30 g over 30 minutes in AGHD patients and in controls, matched for body mass index (BMI), estrogen status, gender, and age. After 43 AGHD patients and 10 controls had been tested, Geref Diagnostic became unavailable in the United States. The study was completed by testing 10 more AGHD patients and 38 controls with macimorelin alone.

### Study drug

Macimorelin was provided by Ardana Biosciences Ltd (Edinburgh United Kingdom) and later by Æterna Zentaris Inc (Basking Ridge, New Jersey). Macimorelin is a ghrelin receptor agonist with a molecular weight of 474.5 g/mol. The solubility in water is 0.3 mg/mL. The structural formula of macimorelin is shown in Supplemental Figure 1, published on The Endocrine Society's Journals Online web site at http://jcem.endojournals.org. GHRH (Geref Diagnostic; Serono) and arginine (Ar-Gine; Pfizer) were purchased from their manufacturers.

### Eligibility criteria

Subjects were 18 years old or older and provided written informed consent. GH deficiency was confirmed by low age- and sex-adjusted IGF-I levels and pituitary hormone deficiencies in 3 or more hormones (TSH, ACTH, LH/FSH, and/or arginine vasopressin) or, for those patients with fewer deficiencies, by one of the following stimulation tests with these cut points for peak GH levels: 1) arginine+GHRH (cut point 4.1 μg/L), 2) ITT (cut point 5.0 μg/L), 3) glucagon stimulation test (cut point 3.0 μg/L), or 4) arginine (cut point 0.4 μg/L) as recommended previously (1, 2, 4, 18). Subjects who required replacement therapy for hormone deficiencies other than AGHD had been on stable treatment for 3 months or longer. Subjects with hypogonadism were on sex steroid replacement therapy, excluding women older than 50 years of age. Controls were matched to AGHD patients already enrolled in the study based on sex; age (±5 years); BMI (±2 kg/m^2^); and estrogen status (women only). They were required to have undergone normal growth and development and have normal prolactin and free T_4_ levels. Females had a history of regular, age-appropriate menses, and males were required to have had normal serum testosterone levels.

Subjects with AGHD were excluded from the study if they had untreated hypothyroidism, intracranial lesions not documented to be stable for 12 months or longer, GH therapy within 1 month of study entry (subjects may have been washed out from previous GH therapy and then screened for other entry criteria), significant cardiovascular or cerebrovascular disease, current active malignancy other than nonmelanoma skin cancer, renal or hepatic dysfunction (≥3 times the upper limit of normal aspartate aminotransferase, alanine aminotransferase, and gamma glutamyl transpeptidase, creatinine > 2 times the upper limit of normal), pregnancy or lactation, active Cushing's disease, or clinically relevant electrocardiogram (ECG) abnormalities (including QT/QTc interval > 450 milliseconds) at any time prior to dosing. Exclusion criteria for the controls included current pregnancy or lactation or clinically relevant ECG abnormalities (including QT/QTc interval > 450 milliseconds).

### Study procedures

Investigators and subjects were not blinded during the study; however, GH assays were performed on blinded samples. In the original protocol, subjects were randomized upon entry into the study to group 1 or group 2, which determined the order of the 2 tests. Half of the patients received macimorelin first, whereas the remaining half received arginine+GHRH first. Each patient's matched control was treated in the same order. In the second phase of the study, subjects received only macimorelin due to the unavailability of Geref Diagnostic (Serono); therefore, the crossover aspect of the design and the randomization no longer applied. All subjects were administered a single oral dose of macimorelin (0.5 mg/kg) in the morning after an overnight fast. (See Supplemental Material for more details.) The GH levels were measured 15–30 minutes before dosing, immediately prior to dosing, and 30, 45, 60, 75, 90, 120, and 150 minutes after dosing. Taste perception was assessed at the time of dosing, and IGF-I was measured before and 150 minutes after dosing. Complete blood count and complete metabolic panel were measured at screening and upon the completion of the study (poststudy visit). In addition, ECGs were also performed prior to dosing and 60 minutes after dosing. For the first portion of the study, the subjects were also asked to rate their preference between the 2 tests.

### Study measures

A central laboratory, Esoterix Clinical Trials (LabCorp) (Cranford, New Jersey), was used to analyze the blood samples. GH levels were measured by a validated immunochemiluminometric assay with an intraassay coefficient of variation of 7.4%, an interassay coefficient of variation of 7%–14%, and a lower limit of quantitation of 0.013 ng/mL. The serum IGF-I levels were measured by a RIA. (See Supplemental Data for more details.)

### Statistical analysis

All statistical analyses were performed using SAS for Windows (version 9.0; SAS Institute, Inc, Cary, North Carolina), MedCalc (version 11.3.1.0; Ostend, Belgium), or Classification and Regression Tree (CART; version 6.0; Statsoft, Tulsa, Oklahoma). Analysis sets reported in the study included per-protocol and safety populations. The per-protocol set (n = 50 cases and 48 controls) included all intent-to-treat subjects who were treated with macimorelin according to the protocol without any major deviations. A protocol deviation was the enrollment of 2 patients who did not meet the entry criteria for confirmed AGHD. Their data were not included in this analysis. The safety population (n = 53 AGHD patients and 48 controls) included all of the subjects who received at least 1 dose of study medication and for whom any safety information was available. The analysis of all safety variables was based on this population.

The primary end point was the area under the receiver-operating characteristic (ROC) curve for the peak GH after macimorelin. The basis for the ROC analysis was the patient or control status of the subject. Secondary efficacy end points included the calculation of Youden's Index for the cutoff with maximum accuracy and the peak IGF-I concentration after treatment. The primary efficacy analysis was the ROC analysis performed on peak GH concentrations when the subjects were treated with macimorelin (see Supplemental Data).

## Results

**Figure 1.**
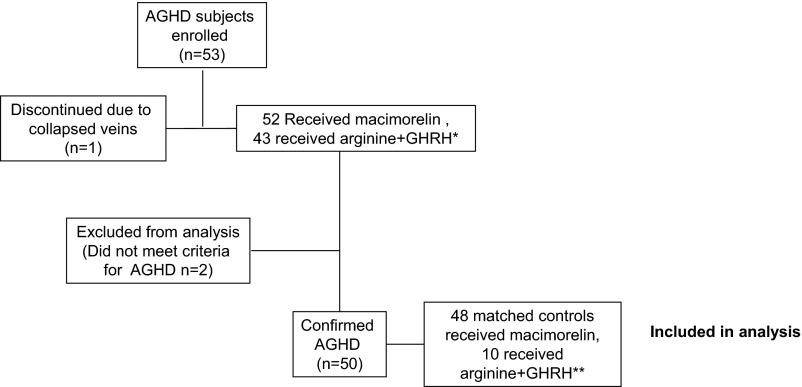
Study design and patient disposition. *, After 43 AGHD patients and 10 controls had been tested, Geref Diagnostic (Serono) became unavailable. The study was completed by testing 10 more AGHD patients and 38 controls with macimorelin alone; **, two AGHD subjects could not be matched due to the combination of young age, high BMI, and estrogen use.

Fifty-three AGHD subjects were enrolled at 11 centers across the United States and 52 received macimorelin. The remaining subject discontinued the study prior to dosing due to collapsed veins after arginine+GHRH. Fifty subjects had confirmed AGHD prior to study entry and were included in this analysis along with 48 controls. Two subjects were excluded from this per-protocol analysis: 1 subject had only 2 pituitary deficiencies instead of the 3 required, and another subject did not have 3 hormone deficiencies and a low IGF-I or confirmed AGHD from an arginine+GHRH test and was randomized in error. Two AGHD subjects could not be matched with a control due to the combination of young age, high BMI, and estrogen use (Figure 1). The 2 groups had similar sex, age, and BMI distribution as designed by the matching criteria, and most subjects were obese. There were more African Americans in the control group. In the AGHD group, 4 subjects were categorized by the investigators as having hypothalamic AGHD, 36 as pituitary AGHD, and 10 as unknown cause. Pituitary adenomas were the most common cause of hormone deficiencies, and within that group nonfunctioning adenomas were noted most often (Table 1). Gonadal, thyroid, and adrenal insufficiency were the 3 most common absent pituitary hormones (83%, 85%, and 59% of AGHD patients, respectively), and sex steroids (87%), T_4_ (85%), and glucocorticoids (62%) were the most commonly used replacement therapies. Surgery (transsphenoidal in 28 of 53 patients, 52.8%; transcranial, 10 patients, 18.9%), medication (7 patients, 13.2%), and radiation (conventional, 8 patients, 15.1%; stereotactic, 5 patients, 9.4%) were the treatments patients had received for their pituitary disorders.

**Table 1.** Demographics and Baseline Characteristics (All Enrolled Subjects)

	AGHD Patients (n = 53)	Matched Controls (n = 48)
Sex, n, %		
Male	31 (58.5)	30 (62.5)
Female	22 (41.5)	18 (37.5)
Race, n, %^a^		
White	49 (92.5)	29 (60.4)
Black or African American	2 (3.8)	18 (37.5)
Asian	2 (3.8)	0 (0.0)
Other	0 (0.0)	1 (2.1)
Ethnicity, n, %		
Hispanic or Latino	11 (20.8)	9 (18.8)
Not Hispanic or Latino	42 (79.2)	39 (81.3)
Age, y, n, %		
Mean (SD)	52 (13.4)	53 (12.9)
Estrogen status, n, %		
None	12 (22.6)	12 (25.0)
Oral	9 (17.0)	6 (12.5)
Patch	1 (1.9)	0 (0.0)
BMI, kg/m^2^, n, %		
Lean (<25)	7 (13.2)	7 (14.6)
Overweight (≥25 and < 30)	15 (28.3)	13 (27.1)
Obese (≥30)	31 (58.5)	28 (58.3)
Mean	32.1 (7.4)	31.8 (7.1)
Etiology, n, %^b^		NA
Pituitary adenoma	34 (64.2)	
CNS tumors	8 (15.1)	
Others^c^	18 (34.0)	

Abbreviation: CNS, central nervous system.

a *P* < .05 between groups.

b More than 1 cause may be applicable per patient.

c Includes 11% head trauma, 4% empty sella, 6% childhood-onset GHD.

### Effect of macimorelin on GH and IGF-I levels

**Figure 2.**
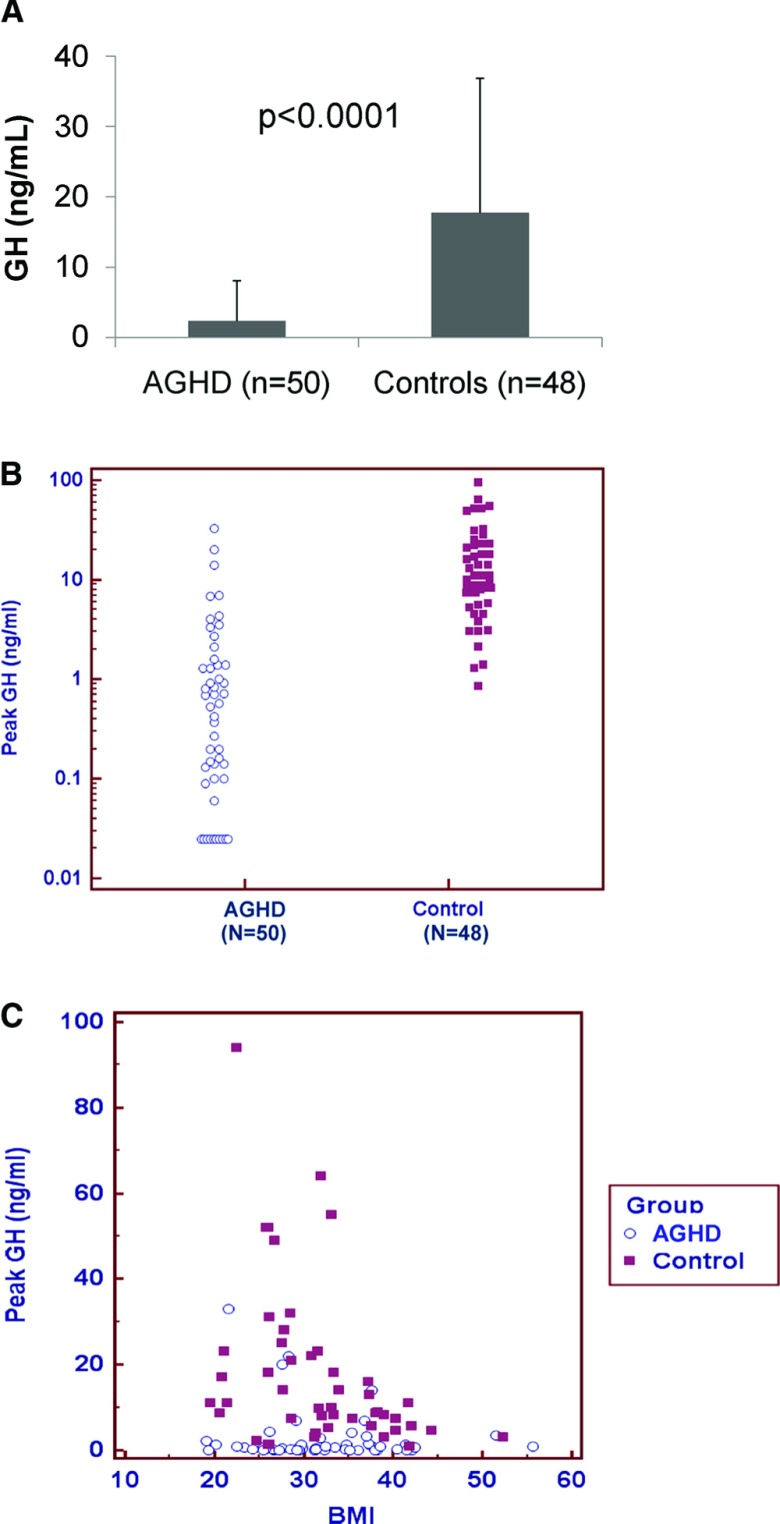
Mean ± SD (A), scatter plot (B) of peak GH concentrations in response to macimorelin and correlation analysis of BMI and peak GH response to macimorelin (C; controls: n = 48, r^2^ = −0.368, *P* = .01; cases: n = 50, r^2^ = −0.14, *P* = .33).

Mean ± SD peak GH levels in AGHD patients and controls after macimorelin administration were 2.36 ± 5.69 ng/mL and 17.71 ± 19.11 ng/mL, respectively (*P* < .0001, Figure 2, A and B). Obesity (BMI > 30 kg/m^2^) was present in 58% of AGHD patients and controls, and peak GH levels were inversely associated with BMI in controls (Figure 2C). As expected, IGF-I levels were lower in the AGHD patients (58.1 ± 37.29 ng/mL) than in the controls (128.1 ± 52.47 ng/mL, *P* < .0001) but did not rise significantly after a single dose of macimorelin (53 ± 34.57 ng/mL and 125.9 ± 54.26 ng/mL respectively). For more details, please see Supplemental Data, Supplemental Tables 1 and 2, and Supplemental Figure 2.

### ROC and CART analysis

**Figure 3.**
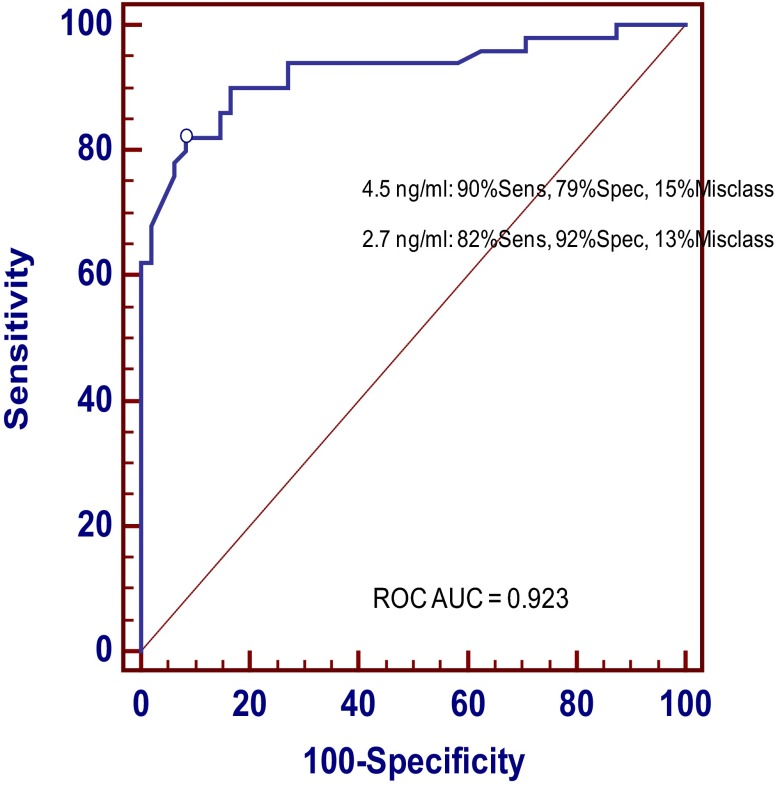
ROC curve and CART for analysis of peak GH in response to macimorelin.

The ROC plot analysis yielded an optimal GH cut point of 2.7 ng/mL, with 82% sensitivity, 92% specificity, and a 13% misclassification rate. A cut point of 4.5 ng/mL yielded a higher sensitivity (90%) but lower specificity (79%) with a misclassification rate of 15% (Figure 3). The CART analysis of peak GH after macimorelin showed that misclassifications of patient and control subjects are slightly decreased when the covariate predictors of BMI and age are included in the analysis. The ROC area under the curve (AUC) and cut point for peak GH alone are slightly different from those found using the logistic regression modeling approach (Supplemental Table 3). When BMI-specific cut points were used on subgroup analyses, the ROC analysis improved, yielding a sensitivity of 86% and a specificity of 92%, with a misclassification rate of 11% (for BMI < 30 kg/m^2^, Youden's cut point 6.8 ng/mL, ROC AUC for BMI < 30 kg/m^2^ = 0.913; for BMI ≥ 30 kg/m^2^, Youden's cut point 2.7 ng/mL, ROC AUC 0.937).

Post hoc analyses were performed to identify the minimum number of blood draws needed to discriminate AGHD patients from controls. Using a GH threshold of 6.8 ng/mL for lean/overweight subjects (BMI < 30 kg/m^2^) and a threshold of 2.7 ng/mL for obese subjects (BMI ≥ 30 kg/m^2^), a sensitivity of 90%, a specificity of 85%, and a 12.2% misclassification rate were attained with a single postdose blood draw at the 45-minute time point. Using time points at 45 and 60 minutes, the sensitivity, specificity, and misclassification rate were 86%, 90%, and 12%, respectively. Using time points at 30, 45, and 60 minutes, the sensitivity, specificity, and misclassification rate were 86%, 92%, and 11%, respectively. The AGHD cases with fewer than 3 deficiencies had a slightly higher mean GH peak value but not significantly different from the cases with at least 3 deficiencies (Supplemental Table 4).

### Macimorelin vs arginine+GHRH test

**Figure 4.**
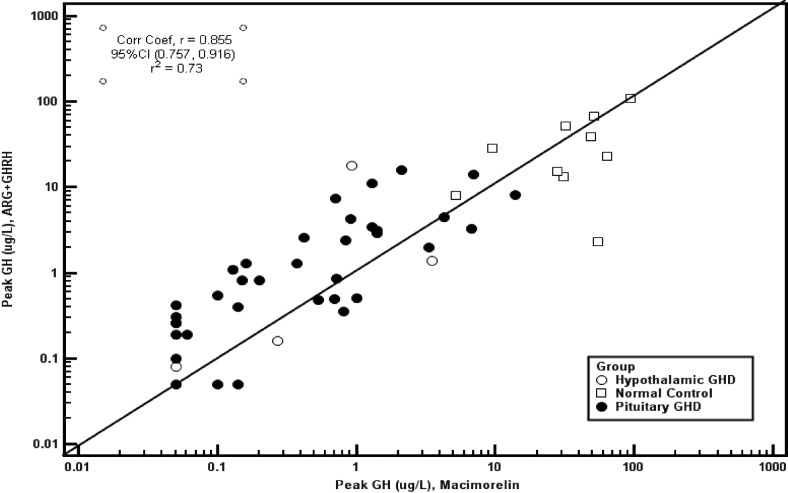
Correlation analysis of peak GH response to macimorelin and arginine+GHRH. The GH detection limit was 0.05 μg/L.

Subgroup analyses of those individuals who underwent both interventions showed that responses were blunted in AGHD patients after arginine+GHRH and macimorelin in comparison with a brisk GH response in normal controls. Receiver operating characteristic curve analysis showed a numerically better discrimination with macimorelin than with arginine+GHRH, but the difference was not statistically significant (*P* = .29). In this subgroup of subjects, macimorelin at peak GH responses of 4.3 μg/L provided a sensitivity of 93%, a specificity of 100%, and a misclassification rate of 6% (ROC AUC 0.99), whereas arginine+GHRH at peak GH responses of 7.4 μg/L showed a sensitivity of 88% with a specificity of 90% and a misclassification rate of 12% (ROC AUC 0.94). As shown in Figure 4, the etiology of AGHD (hypothalamic vs pituitary) did not affect the performance of these tests.

### Taste perception and test preference

Most subjects found the unflavored macimorelin solution to have an unpleasant taste. This was true for both AGHD patients (66.7%) and controls (57.4%). Subjects who did not receive arginine+GHRH (the second portion of the trial) had a better perception of the macimorelin taste (52.1% indicated an unpleasant taste and 41.7% found a neutral taste). Test preference could be evaluated only for the first portion of the trial when subjects received both treatments. In that portion, 72.0% of subjects, significantly more (*P* = .0019) than random choice, preferred the macimorelin test over the arginine+GHRH test.

### Safety and tolerability

Adverse events (AEs) were generally mild or moderate in severity and occurred in 19 of 52 AGHD patients (37%) and in 10 of 48 (21%) controls after macimorelin treatment. In contrast, 26 of 43 AGHD subjects (61%) and 3 of 10 controls (30%) experienced AEs with arginine+GHRH. Only 1 drug-related serious AE was reported in a control subject receiving macimorelin (2.1%), whose postdose ECG showed asymptomatic QT prolongation and nonspecific T wave abnormalities that resolved spontaneously within 24 hours. The AE was rated as serious due to the choice to hospitalize the subject as a precautionary measure. The subject had been taking citalopram, a drug that was later reported by the Food and Drug Administration (FDA) to be associated with QT prolongation (19), although the patient had stopped this medication 7 days prior to dosing (∼ 5 half-lives before the AE).

Among AGHD patients treated with macimorelin, AEs included the unpleasant taste (10 subjects, 19.2%). All other adverse events in the AGHD group treated with macimorelin were reported by a single patient (1.9%) with the exception of diarrhea, which was reported by 2 patients (3.8%). In the matched control group treated with macimorelin, only diarrhea and unpleasant taste were reported by 1 or more subjects (2 subjects each, 4.2%). (See Supplemental Data and Supplemental Table 5 for details.)

## Discussion

Our study demonstrates that a novel oral ghrelin mimetic is both safe and accurate in diagnosing AGHD. The peak GH response after macimorelin treatment allows the establishment of the diagnosis of AGHD with good sensitivity and specificity. Furthermore, this test was well tolerated by patients and does not require parenteral administration of the agent.

Although the ITT is considered the standard reference test for diagnosing AGHD, alternative tests are needed because this test is often contraindicated due to the risks associated with hypoglycemia. In addition, performing an ITT may be challenging in some settings because it requires trained personnel, monitored facilities, and other resources that may not be available to every clinician (2). The arginine+GHRH test had emerged as the best alternative, but unfortunately, Geref Diagnostic (Serono) was removed from the US market in 2008 (1). Other currently available tests such as arginine, clonidine, levodopa, and arginine in combination with levodopa have much lower specificity and sensitivity in adults (1).

Ghrelin and its mimetics are potent GH stimulants, and ghrelin has been used to diagnose GH deficiency in animals and very recently in humans (20, 21). GHRH in combination with the ghrelin mimetic GH-releasing hexapeptide-6 was tested as a method for diagnosing AGHD in one study, and it was found to have excellent sensitivity and specificity with good tolerability (22). Administration of GH-releasing hexapeptide-6 alone was not found useful in the diagnosis of GH deficiency in a different report (23). However, none of these drugs are currently available in the United States.

The current multicenter study of macimorelin was conducted to determine its efficacy and safety in the diagnosis of AGHD. Because other GH stimulation tests require iv or im administration of the GH stimulant, macimorelin offers the advantage of being administered orally. In addition, the drug is rapidly absorbed and has good bioavailability, reaching peak serum concentrations 45 minutes after administration (17). Although the unavailability of Geref Diagnostic (Serono) to complete the protocol as initially designed greatly decreased the statistical power of the study to detect a significant difference in the accuracy of the macimorelin vs the arginine+GHRH test in diagnosing AGHD, the results on the subgroup of subjects whom we could test with both agents show that macimorelin is at least as accurate in diagnosing this condition as arginine+GHRH, and it is preferred by patients.

A single blood draw at 45 minutes after the dose showed very good sensitivity, specificity, and misclassification rate that was only marginally improved by addition of the 30- and 60-minute blood draws. However, in the clinical setting, providers may choose to perform these 3 draws (30, 45, and 60 minutes) to maximize the chance of a correct diagnosis because missed specimens are not uncommon. This test would also present several advantages over other current alternatives such as the glucagon stimulation test. Only 3 draws are needed after drug administration without the need to follow up the patients for extended periods of time, it does not require parenteral administration of drugs (although it has to be diluted in water before administration), and it is not associated with nausea or symptomatic hypoglycemia.

Peak GH levels in response to macimorelin were inversely related to BMI in control subjects. This is important, given that obesity affects more than 30% of the adult US population and the specificity of GH provocative tests may be decreased in this setting (24, 25). Others have suggested that BMI-specific cut points should be used for GH-provocative tests (26). Our data show that the sensitivity of the macimorelin-stimulated GH test can be improved while maintaining specificity by using different cut points based on BMI level.

Macimorelin was well tolerated in our study. The only adverse event reported frequently was dysgeusia (unpleasant taste). Nevertheless, most subjects preferred this test over the arginine+GHRH test. Only 1 subject experienced a serious adverse event of asymptomatic QT prolongation and T wave abnormality on the ECG that resolved overnight. To our knowledge, there are no prior published reports of this or other drugs in this class causing ECG abnormalities. It is worth mentioning that the individual that experienced this AE had been taking citalopram, a drug now known to cause QT prolongation (19). Other known effects of activating the ghrelin receptor including increased appetite and body weight were not seen and are unlikely to happen with a single dose.

There are several strengths to our study. The use of BMI-, estrogen status-, and age-matched controls decreased the chances of misclassifying individuals due to variability in these variables. Also, a central reference laboratory was used to measure the GH levels. Limitations of our study include the fact that we were not able to perform the arginine+GHRH test in all subjects as originally planned because Geref Diagnostic (Serono) became unavailable during the study and that there was some overlap between AGHD patients and controls. Nevertheless, the sensitivity and specificity thresholds determined by this study were similar to those reported previously for other GH provocative tests (1). Also, the limited number of patients did not allow for a cause-specific (hypothalamic, n = 4, vs pituitary, n = 36) analysis of the data or for further analyses based on other patient features (ie, 3 confirmed hormone deficiencies vs less than 3 hormone deficiencies and a positive confirmatory test). Subjects with diabetes, renal, or hepatic dysfunction also were excluded from this trial. Further studies including a larger number of these patients will be needed to determine the sensitivity and specificity of this test in these scenarios. It is possible that the combination of arginine and macimorelin will cause a greater release of GH than macimorelin alone, given that arginine is thought to potentiate the response to GHRH via inhibition of hypothalamic somatostatin release (27). Future studies also will be needed to address this question.

There was a difference in cut points for macimorelin-stimulated peak GH levels for those controls receiving both tests vs the entire cohort. Several factors could have contributed to this difference, including chance differences due to the small sample size of the first cohort (n = 10). The use of this agent to discriminate those patients with hypothalamic disease from pituitary disease including patients with recent irradiation or tumors in the hypothalamus such as hamartomas remains to be seen. Arginine+GHRH is associated with false-normal responses in these circumstances. It may turn out that patients with hypothalamic disease patients will not respond to this agent and provide an important discriminating tool. Further studies using a larger sample size would be needed to definitively answer this question, although this would be possible only if Geref Diagnostic (Serono) becomes available again. Unpleasant taste was the only adverse event reported frequently with macimorelin. Alternative strategies to improve its palatability including using a diluent other than water are being tested in ongoing trials.

In conclusion, this study shows that oral macimorelin is safe and effective in diagnosing GH deficiency in adults, with sensitivity and specificity comparable with other provocative tests. This novel oral test agent could be a simple, rapid, and convenient alternative, especially for patients in whom ITT is contraindicated in establishing the diagnosis of AGHD that could be performed in most outpatient settings.
